# Morphological and Functional Remodeling of the Ischemic Heart Correlates with Homocysteine Levels

**DOI:** 10.3390/jcdd10030122

**Published:** 2023-03-14

**Authors:** Attila Cziraki, Zoltan Nemeth, Sandor Szabados, Tamas Nagy, Márk Szántó, Csaba Nyakas, Akos Koller

**Affiliations:** 1 Heart Institute, Medical School and Szentágothai Research Centre, University of Pecs, 7624 Pecs, Hungary; cziraki.attila@pte.hu (A.C.);; 2 Department of Morphology and Physiology, Faculty of Health Sciences, Semmelweis University, 1088 Budapest, Hungary; 3 Eötvös Loránd Research Network, Semmelweis University (ELRN-SU), Cerebrovascular and Neurocognitive Disorders Research Group, Department of Translational Medicine, Faculty of Medicine, Semmelweis University, 1094 Budapest, Hungary; 4 Department of Laboratory Medicine, Medical School, University of Pecs, 7624 Pecs, Hungary; 5 Research Center for Sports Physiology, Hungarian University of Sports Science, 1123 Budapest, Hungary; 6 Department of Physiology, New York Medical College, Valhalla, NY 10595, USA

**Keywords:** myocardial ischemia, remodeling, cardiac marker, inflammation, pericardial fluid, homocysteine, troponin-I

## Abstract

Background: Homocysteine (Hcy) is involved in various methylation processes, and its plasma level is increased in cardiac ischemia. Thus, we hypothesized that levels of homocysteine correlate with the morphological and functional remodeling of ischemic hearts. Thus, we aimed to measure the Hcy levels in the plasma and pericardial fluid (PF) and correlate them with morphological and functional changes in the ischemic hearts of humans. Methods: Concentration of total homocysteine (tHcy) and cardiac troponin-I (cTn-I) of plasma and PF were measured in patients undergoing coronary artery bypass graft (CABG) surgery (*n* = 14). Left-ventricular (LV) end-diastolic diameter (LVED), LV end-systolic diameter (LVES), right atrial, left atrial (LA) area, thickness of interventricular septum (IVS) and posterior wall, LV ejection fraction (LVEF), and right ventricular outflow tract end-diastolic area (RVOT EDA) of CABG and non-cardiac patients (NCP; *n* = 10) were determined by echocardiography, and LV mass was calculated (cLVM). Results: Positive correlations were found between Hcy levels of plasma and PF, tHcy levels and LVED, LVES and LA, and an inverse correlation was found between tHcy levels and LVEF. cLVM, IVS, and RVOT EDA were higher in CABG with elevated tHcy (>12 µM/L) compared to NCP. In addition, we found a higher cTn-I level in the PF compared to the plasma of CABG patients (0.08 ± 0.02 vs. 0.01 ± 0.003 ng/mL, *p* < 0.001), which was ~10 fold higher than the normal level. Conclusions: We propose that homocysteine is an important cardiac biomarker and may have an important role in the development of cardiac remodeling and dysfunction in chronic myocardial ischemia in humans.

## 1. Introduction

Ischemic heart disease, including coronary artery disease, is the leading cause of death worldwide [1]. Impaired coronary blood supply develops due to narrowing and/or constriction of coronary vessels of different sizes [2]. Myocardial ischemia causes injuries to the myocardium and simultaneously initiates compensatory mechanisms in the injured tissues, increases de novo protein synthesis and connective/fibrotic tissues, leading to a structural remodeling and functional changes of the heart [3,4,5]. Cardiac remodeling involves several signaling mechanisms, including, among others, DNA methylation, nitric oxide/asymmetric dimethyl-arginine (NO/ADMA) pathway, endothelin-1, and angiotensin II [6,7,8,9]. Methionine, an essential amino acid, plays an important role by providing methyl groups for protein and DNA methylation pathways. It is converted to s-adenosylmethionine, a general methyl donor in the cells, which provides methyl groups in trans-methylation reactions, for example, to the synthesis of methylarginines.

Homocysteine (Hcy) is a sulfur-containing amino acid, which is metabolized via re-methylation by converting Hcy back to methionine, and trans-sulfuration by converting Hcy to cysteine and taurine amino acids [10]. Although the normal plasma level of Hcy can be disputed, in healthy young subjects, values above 15 µM/L are considered to be high, and the optimal homocysteine levels are thought to be below 10–12 µM/L [11,12,13,14,15].

Despite earlier reports, as of today, there is still no consensus regarding the reference limits for plasma homocysteine levels. Studies focusing on various parts of the population suggest that the upper limit of 15 µmol/L is too high in normally nourished people without vitamin deficiencies. Additionally, it seems that each 5 µmol/L increase in Hcy level increases the risk of CHD (independently of other traditional risk factors) by about 20% and that a continuum exists with the subsequent risk. Some reports suggest that a level around 6 µmol/L Hcy should be considered normal [16]. A systematic review and meta-analysis of homocysteine level and coronary heart disease incidence are presented in [16]. Thus, as Milani and Lavie posited, “homocysteine, however, remains an important field of study as an unconventional risk factor, one facet of a complex metabolic puzzle—a veritable Rubik’s cube—that promotes atherosclerosis” and cardiac dysfunction [17].

Indeed, elevated plasma levels of Hcy play an important role in the development of cardiovascular diseases, such as coronary artery disease and atherosclerosis, which may further exacerbate cardiac ischemia/infarction and fibrosis [18,19]. Supporting this idea, previous studies by Jacob et al. have shown that hyperhomocysteinemia leads to pathological ventricular hypertrophy in normotensive rats [20]. However, few if any data are available in humans on whether or not plasma (PL) and pericardial fluid (PF) Hcy correlates with ischemic cardiac remodeling. 

Cardiac ischemia in humans—in many cases—is resolved by successful coronary bypass graft surgery (CABG) [21]. Because from the ischemic/injured cardiac muscle cardiac troponin-I (cTn-I) is released and thus becomes elevated in the plasma, it is used as one of the biomarkers/indicators of ischemic insult to the cardiac muscle [22,23,24]. Interestingly, its level, like the level of Hcy in the PF, has not yet been measured.

Based on the aforementioned facts, we hypothesize that the levels of Hcy in the plasma and pericardial fluid correlate with cardiac remodeling of the ischemic heart of patients undergoing CABG treatment. Thus, we collected samples of the plasma and PF of patients undergoing CABG surgery and measured their total Hcy and cTn-I concentrations and correlated them with the morphological and functional characteristics of their hearts.

## 2. Materials and Methods

### 2.1. Study Description and Clinical Characterization of the Patients

In the present study, subjects were recruited at the Heart Institute of the University of Pécs Medical School, Hungary. This is a cross-sectional investigation of 14 subjects with coronary artery disease and 10 non-cardiac patients (NCP) as a control for echocardiographic measurements. Non-cardiac patients underwent physical examination with no cardiac surgery. All patients with coronary artery disease underwent elective CABG surgery. Age- and sex-matched subjects with mild to moderate arterial hypertension were selected for the study. Written informed consent was obtained from all individuals before participation in the study. The investigation and consent documents were approved by the Ethics Committee of the Medical School of the University of Pecs (RKEB-4123/20110). The investigation conforms to the principle outlined in the Declaration of Helsinki. Blood plasma and PF samples were collected from the patients after median sternotomy.

### 2.2. Measurements of tHcy and cTn-I 

Pericardial fluid samples were collected by pericardiocentesis, together with blood samples, simultaneously from CABG patients in heparinized vacutainer tubes during CABG surgery, and centrifuged at 3000 rpm for 10 min. Supernatants were then kept at –80 degrees for further use. cTn-I was measured by Microparticle Enzyme Immunoassay, and tHcy was measured by Fluorescence Polarization Immunoassay on an Abbott Axsym immunochemical automated analyzer (Abbott Diagnostics, Abbott Laboratories, Abbott Park, IL, USA), according to the manufacturer’s instructions [25,26].

### 2.3. Echocardiography Measurements

Morphological characteristics of CABG patients’ hearts were assessed with 2-D transthoracic echocardiography. Two-dimensional (2-D), M-mode, and Doppler echocardiography with automated border detection were carried out using Hewlett-Packard Sonos 5500 echocardiograph with a 2.5 MHz transducer (Hewlett-Packard, USA). Two-dimensional echocardiographic measurements were performed according to European guidelines. In order to minimize the variability and bias, echocardiographic measurements were carried out by the same cardiologist (blinded to the patient’s identity) with an expert license in transthoracic echocardiography. All images were recorded and analyzed offline. The following parameters were measured: left ventricular end-diastolic diameter (LVED), left ventricular end-systolic diameter (LVES), the thickness of interventricular septum (IVS) and posterior wall (PW), right ventricular outflow tract end-diastolic area (RVOT EDA), and right atrial (RA) and left atrial (LA) area. The biplane Simpson method using the end-diastolic and end-systolic apical 4- and 2- chamber views for estimation of LV volume and calculation of the ejection fraction (LVEF) [27] was applied.

### 2.4. Statistics and Calculations

Correlations between tHcy levels and echocardiographic parameters were performed using Pearson’s correlation analysis. Because we found a very high correlation between PF and plasma values of Hcy for correlation analysis plasma and PF, tHcy levels (plasma + PF)/2 were averaged. Plasma and PF tHcy and cTn-I levels were compared with a two-tailed independent *t*-test. For comparison of echocardiographic parameters of CABG patients with non-cardiac patients (NCP), two-tailed independent *t*-test was applied. Left ventricular mass (LVM) was calculated using the American Society of Echocardiography (ASE) convention: VM = 0.8 (1.04 ([LVED + PW+ IVS]3- [LVED]3)) + 0.6 g, where PW is posterior wall thickness [28]. Statistically significant changes were considered at *p* < 0.05.

## 3. Results

### 3.1. Characteristics of Patients

Descriptive statistics of the patients are summarized in Table 1, showing the major demographic and clinical characteristics, as well as concomitant risk factors and medications of patients. Six patients exhibited high blood pressure, and seven patients exhibited left-ventricular hypertrophy (LVH). The types and number of CABG operations were as follows: CABGx2: 1: CABGx3: 10; CABGx4: 3.

**Figure 1:**
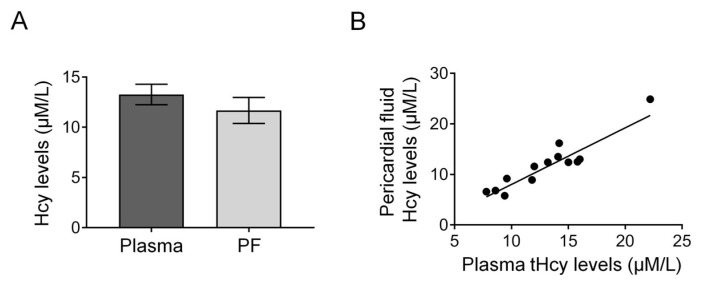
Data show that homocysteine levels of the plasma and pericardial fluid (PF) are similar and correlate to each other in patients undergoing CABG surgery (*n* = 14). Two-tailed independent *t*-test showed no difference between plasma and PF tHcy levels (*p* > 0.05) (**A**). Pearson’s correlation analysis showed a positive correlation between plasma and PF levels of CABG patients (**B**). y = 1.121x − 3.183, r = 0.9, *p* < 0.0001. PF—pericardial fluid, tHcy—total homocysteine; Data expressed as mean ± SEM; *p* < 0.05 was considered significant.

Homocysteine levels in the plasma and PF were similar and showed positive correlation in CABG patients. We found that mean tHCy levels were similar in the PF and plasma (PF vs. plasma: 11.5 ± 1.26 μM/L vs. 13.4 ± 1.04 μM/L, *p* = 0.2628) (Figure 1A). In addition, we found a positive correlation between PF and plasma tHcy levels in this group (r = 0.9, *p* < 0.0001) (Figure 1B).

### 3.2. Echocardiographic Parameters of the Heart in CABG Patients

**Figure 2:**
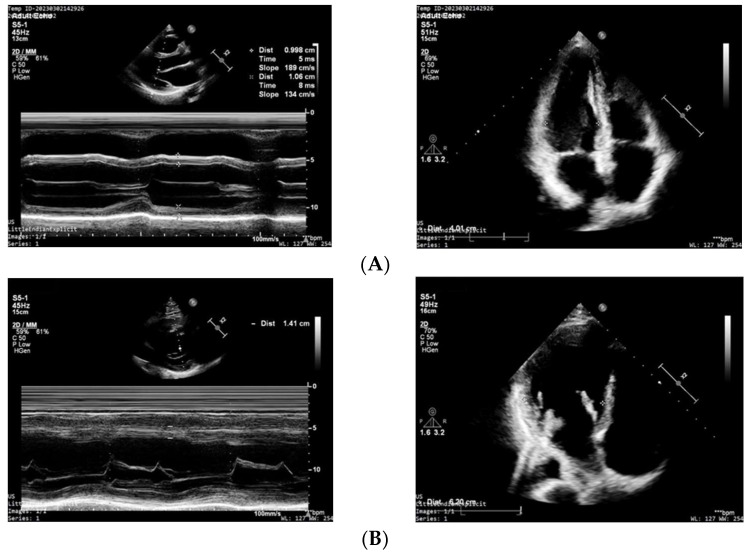
(**A**) Two-dimensional transthoracic echocardiographic image of a CABG patient with tHcy < 12 μM/L. Left panel: parasternal long axis view: normal thickness of interventricular septum (IVS) and posterior wall (PW) can be seen (10 mm). Right panel: Apical four-chamber view shows a normal left-ventricular end-diastolic diameter (LVED = 40 mm). (**B**) Two-dimensional transthoracic echocardiographic image of a CABG patient with tHcy > 12 μM/L. Left panel: Parasternal long axis view: considerably increased (14 mm) thickness of interventricular septum (IVS) and posterior wall (PW) can be seen. Right panel: Apical four-chamber view shows an increased left-ventricular end-diastolic diameter (LVED = 60 mm).

According to previous a publication [11] the optimal level of Hcy is considered to be below 12 µM/L; thus, we grouped CABG patients with tHcy levels below or above 12 µM/L [11]. In Figure 2, representative echocardiographic images of a CABG patient with tHcy < 12 μM/L (A) and a CABG patient with tHcy > 12 μM/L Hcy (B) can be seen.

We found that the LVED and LVES of CABG patients with tHcy higher than 12 µM/L were greater than that of lower tHcy. The LA of CABG patients with elevated tHcy is higher than that of CABG patients with lower tHcy. In CABG patients with elevated tHcy, the cLVM was higher than that of CABG patients with lower tHcy. The LVEF, PW, and RA were similar between the two groups (Table 2).

### 3.3. Correlation of Echocardiographic Parameters of the Heart with Homocysteine Levels in CABG Patients

**Figure 3:**
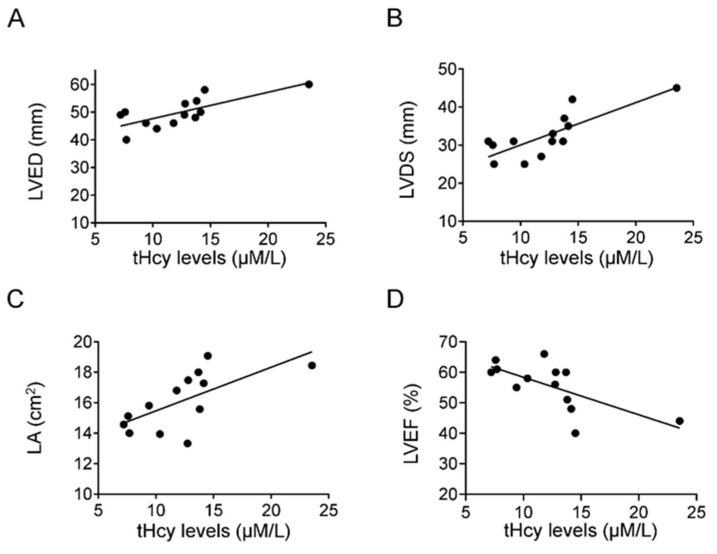
Data show that echocardiographic parameters of the heart correlate with homocysteine levels of patients undergoing CABG surgery. Pearson’s correlation analysis showed positive correlations between tHcy levels vs. LVED (y = 0.8499x + 38.76; r = 0.6; *p* < 0.01), LVDS (y = 0.9713x + 19.82; r = 0.7; *p* < 0.01), LA (y = 0.2956x + 12.54, r = 0.7, *p* < 0.01; **A**–**C**), and an inverse correlation between tHcy levels vs. LVEF (y = −1.0154x + 69.299; r = 0.5; *p* < 0.05) (**D**). CABG—coronary artery bypass graft surgery; LVED—left ventricular end-diastolic diameter; LVDS—left ventricular end-systolic diameter; LA—area of the left atrium; LVEF—left-ventricular ejection fraction; tHcy—total homocysteine; *p* < 0.05 was considered significant.

Analyzing correlations of homocysteine levels and echocardiographic parameters revealed meaningful information. We found that tHcy levels positively correlated with LVED (r = 0.6, *p* < 0.01) (Figure 3A), LVDS (r = 0.7, *p* < 0.01) (Figure 3B), and LA (r = 0.7, *p* < 0.01) (Figure 3C) and inversely correlated with LVEF (r = 0.5; *p* < 0.05) (Figure 3D).

### 3.4. Cardiac Troponin-I in Levels Are Increased in PF of CABG Patients

**Figure 4:**
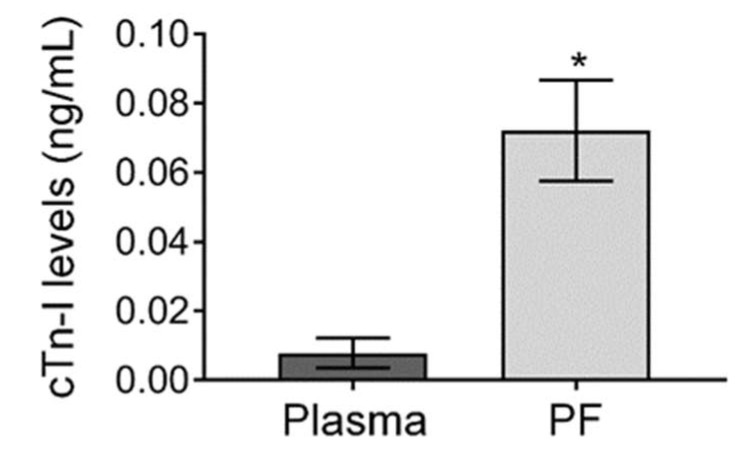
Data show that in CABG patients, compared to the plasma level, cTn-I level is significantly greater in the pericardial fluid (PF). Two-tailed independent *t*-test showed that cTn levels of PF were significantly higher than that of blood plasma (0.01 ± 0.004 vs. 0.07 ± 0.01 ng/mL, respectively, *p* < 0.05). PF—pericardial fluid, cTn-I—cardiac troponin-I. Data expressed as mean ±SEM. *p* < 0.05 was considered significant, which is indicated with an asterisk.

We have found that troponin-I levels were significantly higher in the PF than the plasma of CABG patients indicating myocardium hypoxia and injury (PF vs. plasma: 0.08 ± 0.02 ng/mL vs. 0.01 ± 0.003 ng/mL, *p* < 0.001) (Figure 4).

## 4. Discussion

The salient findings of the present study are as follows: (1) There is a positive correlation between plasma and pericardial fluid Hcy levels. (2) The echocardiographic parameters, namely, end-diastolic (LVED), end-systolic diameter of the left ventricle (LVDS), the left atrial (LA) area, calculated left ventricular mass (cLVM), right ventricular outflow tract end-diastolic area (RVOT EDA), and thickness of the interventricular septum (IVS) were significantly higher in CABG patients with tHcy above 12 μM/L compared to non-cardiac patients. (3) There are positive correlations between tHcy levels and the structural changes in LVED, as well as LVDS, LA, and an inverse correlation between tHcy levels and left-ventricular ejection fraction (LVEF) in CABG patients. (4) The level of cardiac troponin-I was significantly higher in pericardial fluid than plasma.

### 4.1. Cardiac Ischemia, Remodeling and Homocysteine 

CABG surgery is a widely used surgical solution for myocardial ischemia due to infarction or narrowing of the large coronary arteries. The persistent myocardial ischemia before CABG surgery causes injury to the myocardium, initiating simultaneous compensatory mechanisms that lead to changes in the size of cardiac chambers (Figure 2). Indeed, we found an increase in the end-diastolic and systolic diameter (Table 2). In addition, we also found an increase in the thickness of IVS, calculated LVM, and right ventricular outflow tract end-diastolic area (RVOT EDA) (Figure 2, Table 2). These findings suggest that there is a hypertrophic remodeling in this group of patients, which may correspond to the increased level of Hcy in the plasma and PF. This idea is supported by the close correlation between Hcy level and morphological changes in the present study (Figure 3) and that of previous animal and human studies, indicating that elevated plasma Hcy levels are associated with cardiac ischemia and remodeling [29,30].

Plasma tHcy level considered to be normal between 5–15 μmol/L, and its elevation can contribute to coronary artery disease in humans [11,12]. Higher levels of Hcy—in addition to remodeling—have functional consequences, as shown by the reduced ejection fraction, suggesting contractile dysfunction (Figure 3D). An interesting novel finding of the present study is that the tHcy levels of PF and plasma are similar, and there is a positive correlation between the plasma and PF tHcy levels (Figure 1), suggesting that homocysteine can freely diffuse between the coronary vessels and cardiac interstitial space, thereby reaching the pericardial space. This could be explained by the small size of the Hcy molecule and by the inflammation-induced increased permeability of the epicardium.

### 4.2. Presence of Cardiac Hypoxia in the Patient Group Studied

Cardiac troponins have been found to have high sensitivity as indicators of myocardial injuries, such as in myocardial ischemia. Cardiac troponin level in the plasma is <0.004 ng/mL or <0.005 ng/mL [23]. In the present study, we found that cTn-I levels were approximately 10-fold higher than the normal level and in the PF were significantly higher than in the plasma (Figure 4). This indicates that the origin of PF cTn-I is the cardiac tissues, explaining the injury of the myocardium, and thus, it has a higher diagnostic value than that of plasma. The upper limit for high sensitivity cTn-I was reported to be <0.004 ng/mL or <0.005 ng/mL [23] in the plasma; however, cardiac troponin assays are regarded as a biomarker for detecting acute myocardial necrosis, but they may also be released in the absence of cardiac necrosis [24]. 

### 4.3. Previous Findings with Homocysteine and the Heart

The Framingham Heart Study showed that Hcy levels in the plasma of cardiac patients are positively related to changes of the left ventricular structure and function, such as left ventricular wall thickness [31]. In the present study, we found positive correlations between the chambers of the left side of the heart and PF tHcy levels in CABG patients (Figure 3). This suggests that Hcy may contribute to cardiac remodeling in the ischemic heart. This conclusion is supported by experimental findings in rat by Chen et al. [32].

Interestingly, we have previously found in rats with elevated homocysteine that increases in flow elicit constrictions of isolated arterioles, instead of dilation, which were attributed to enhanced production of reactive oxygen species known to decrease the bioavailability of nitric oxide (NO).The lack of NO could contribute to hypoxia and inflammation [30,33], and also, —being an antigrowth factor—could contribute to cardiac remodeling.

### 4.4. Pathomechanisms That May Contribute to Cardiac Remodeling and Contractile Dysfunction: Human Pericardial Fluid ADMA and Endothelin and Cardiac Ischemia

Previously, we found elevated asymmetric dimethylarginine (ADMA) levels in cardiac ischemic and valve diseased patients, which showed positive correlation with indices of cardiac hypertrophy [6]. We have also shown that in the pericardial fluid, several biologically active substances are present, such as endothelin-1, the increased level of which contributes to pathological cardiac function [34]. In addition, cardiac ischemia can initiate inflammatory responses—mediated by immune cells and inflammatory cytokines—eliciting adaptation to hypoxic conditions by cellular hyperplasia or to cell death by apoptosis or necrosis. The underlying molecular signaling of higher Hcy level is likely to involve several parallel running events, such as cardiac ischemia, oxidative stress, and inflammation, leading to an increased level of ADMA levels known to enhance the growth factor angiotensin II by activating the tissue renin–angiotensin system’s [35] cell apoptosis and necrosis, and then initiating—under these conditions—the dysmethylation of proteins and genes, all of which are likely to be responsible for cardiac muscle remodeling and dysfunction [36].

### 4.5. Hypoxia and Inflammation May Contribute to Cardiac Remodeling and Contractile Dysfunction

Hypoxia and inflammation can initiate fibrotic processes in which the heart undergoes structural remodeling, with consequent functional changes [37]. The initial mechanism for cardiac fibrosis is the fibroblast-to-myofibroblast transition, in which cardiac fibroblasts become activated and converted into myofibroblasts [38]. Myofibroblasts are characterized by the expression of α-smooth muscle actin and increased production of collagens, as well as the capability to contract [39,40]. During the process of cardiac fibrosis, myofibroblasts secrete excessive collagens in their extracellular matrix (ECM) and finally undergo apoptosis resulting in irreversible fibrosis [40]. Inflammatory signals, such as transforming growth factor beta (TGF-β), activate cardiac fibroblasts, while non-coding RNA transcripts, such as microRNAs (miRNAs), mediate gene regulation during cell transition [41]. This process involves epigenetic mechanisms, such as DNA methylation, and post-translational protein modifications, which regulate the myofibroblast phenotype in fibrosis [42]. Simultaneously, post-translational protein modifications’ methylation processes are increased during cardiac remodeling, which are carried out by methyltransferases [43], for instance, protein-arginine methyltransferase-1 methylates arginine forming asymmetric dimethylarginine, by which the methyl donor S-adenosylmethionine is utilized, converting to S-adenosylhomocysteine. In this process, S-adenosylhomocysteine is converted back into S-adenosylmethionine or more likely into adenosine and Hcy. The sulfur-containing amino acid Hcy is a crucial player in trans-methylation processes [10].

All of these molecular pathomechanisms can lead to changes in cardiac substrate utilization [44], reduced contractility, and eventually heart failure. Supporting this idea, Okuyan et al. reported that NT-proBNP, hs-CRP, E/A ratio, and HbA1C were independently associated with hyperhomocysteinemia in a patient with diastolic heart failure. [45].

**Figure 5:**
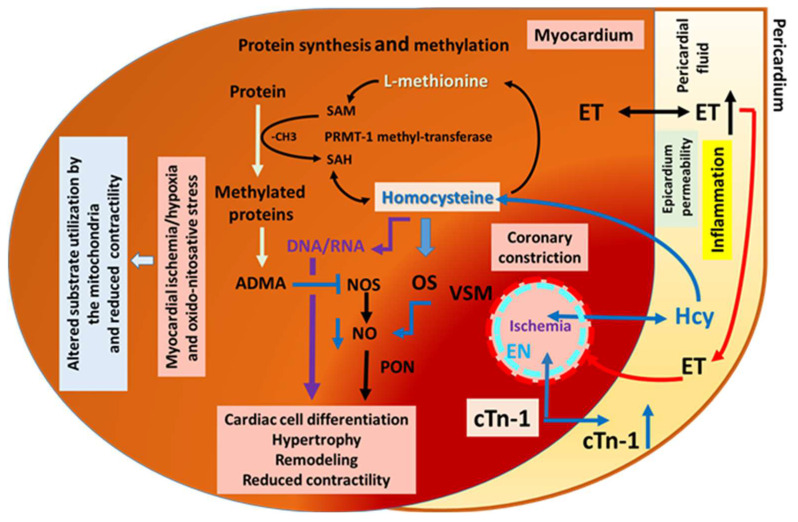
Summary of some of the mechanisms involved in the pericardial homocysteine-related cardiac remodeling and coronary dysfunction. SAM: S-adenosylmethionine, SAH: S-adenosylhomocysteine, ADMA: asymmetric dimethylarginine NOS: nitric oxide synthase, PON: peroxynitrite, ET: endothelin.

In Figure 5, we summarized some of the main mechanisms of action of homocysteine, which can lead to functional and morphological cardiac remodeling and microvascular constrictions due to the free movement of endothelin in the pericardial fluid/sac, reaching remote cardiac muscle areas.

### 4.6. Clinical Importance of the Present Findings

**Figure 6:**
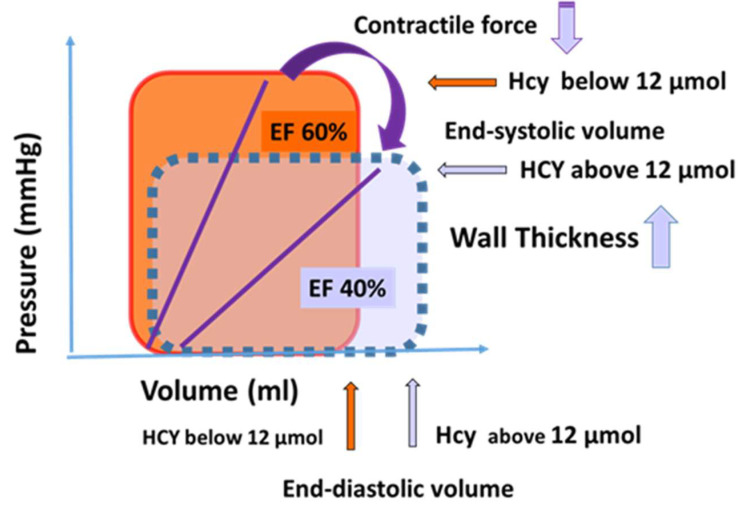
On the basis of our findings, we developed hypothetical cardiac cycles indicating the differences in the cardiac function of the two groups of patients with lower or higher levels of homocysteine.

Our data suggest that it is important to measure homocysteine level in patient populations with ischemic heart disease, because higher but still normal levels of homocysteine may represent risk factors for morphological and functional remodeling of the heart in this condition. On the basis of our findings in Figure 6 we are showing two hypothetical cardiac cycles curves indicating that in a condition of higher homocysteine level end-diastolic volume increases, contractility decreases, resulting in a reduced ejection fraction.

The higher level of Hcy may be due to—among other factors—low vitamin B6, B12, and folate levels, which, however, can be corrected with appropriate therapy in most cases [46]. The normal range for Hcy (like many other parameters) usually refers to young (25-year-old) healthy individuals. However, in diseased conditions, the normal—or, as we can call, optimal—range of homocysteine may shift to higher or lower values/range. For example, it is thought that systemic blood pressure in diabetic patients should be lower than in non-diabetics [47]. Thus, it is possible that in cardiac ischemia, reducing Hcy levels below the “normal” range is beneficial to prevent cardiac remodeling and fibrosis. Since the level of Hcy can be influenced by appropriate treatment, it could be recommended to do so in ischemic heart disease. The pericardial fluid Hcy and cardiac troponin levels could be important biomarkers of cardiac ischemia when their plasma levels are still in the normal range. 

### 4.7. Clinical Aspects Related to Medications

It is of note that the long-term administration of glucose-lowering drugs such as metformin may attenuate cardiac hypertrophy and improve cardiac function, as suggested by clinical and animal studies [48,49,50]. However, studies have also shown that anti-diabetic drugs could increase plasma homocysteine levels [51]. In the present study, 6 out of 14 patients had diabetes and received anti-diabetic drugs (such as metformin or Levemir); however, the LVM values of these six patients were similar to those of patients with no anti-diabetic medication. We also found that the tHcy levels of patients with diabetes were similar to those with no diabetes, suggesting that these differences did not significantly affect the morphological findings in these groups of patients. Another important aspect is that plasma lipid levels may influence cardiac morphology and function. Indeed, a recent clinical study showed a positive correlation between higher LDL cholesterol and higher LV end-diastolic volume, as well as higher LVM [52]. Among our patients, only one had borderline high total cholesterol and high triglyceride level, and both the LVM and LVEF values of this patient were in the average range. Additionally, it is known that cholesterol-lowering drugs, e.g., statins, inhibit cardiac hypertrophy [53]. In the present study, all patients but one received statins, and half of the patients exhibited left-ventricular hypertrophy, but their other cardiac parameters were similar. Likewise, there was a consistent correlation between Hcy levels and the echocardiographic parameters, suggesting that statins were unlikely to have influenced the findings of the present study. Thus, cardiac morphology and function of patients included in the present study were unlikely to be affected by glucose-lowering drugs or plasma cholesterol levels.

#### Limitations of the Study

As in most human studies, there are no appropriate controls; thus we used findings from experimental (animal) studies to draw our conclusions regarding the underlying mechanisms. In this human study, we were limited by the amount of coronary bypass graft surgery (CABG) performed at our institution. Due to the great advances in percutaneous intervention (PCI), fewer patients need this type of open chest surgery. In addition, we were limited regarding the number of patients available in our clinic, which fall into the category of our investigation. Nevertheless, the homogeneity of patients allowed for the statistical analysis of data. For this reason, more data are necessary to support our conclusions regarding the role of homocysteine metabolism in cardiac remodeling due to long-lasting ischemia. In addition, it is of note that the anti-diabetic medication may influence the homocysteine levels of these CABG patients [54].

## 5. Conclusions

In conclusion, the findings of the present investigation suggest that in chronic ischemic cardiac patients—as indicated by the increased troponin level in the pericardial fluid—the higher levels of homocysteine (but still in the “normal range”) in the pericardial fluid and plasma contribute to the development of cardiac remodeling and contractile dysfunction. Thus, in this special patient population, plasma homocysteine levels should be measured, considered, and lowered with appropriate therapy.

## Figures and Tables

**Table 1:** Pre-operative characteristics and medications of CABG patients. Data are expressed as means ± SEM. Data were compared using 2-tailed independent *t*-test. GABG—coronary artery bypass graft surgery; LVH—left-ventricular hypertrophy.

Pre-Operative Data	
Age (year)	62.1 ± 2.1
Sex (male/female)	7/7
Hypertension	6
Echocardiographic indices of LVH	7
Diabetes mellitus	6
Previous acute myocardial infarction	2
**Pre-operative medication**	
Beta-blocker	13
Ca-channel blocker	2
ACE-inhibitor	5
Diuretic	1
Aspirin	12
Antiplatelet medication (Clopidogrel)	6
Anti-diabetic	6
Statin	13
Anti-arrhythmic	2

**Table 2:** Echocardiographic data of cardiac patients CABG with tHcy < 12 μM/L, and CABG tHcy > 12 μM/L.

Echocardiographic Parameter	CABG tHcy < 12 μM/L	CABG tHcy > 12 μM/L	*p*
LVED (mm)	45.83 ± 1.47	52.00 ± 1.88	*p* < 0.05
LVES (mm)	28.17 ± 1.17	34.75 ± 2.35	*p* < 0.05
LA (cm^2^)	14.71 ± 0.68	17.12 ± 0.65	*p* < 0.05
LVEF (%)	60.67 ± 1.63	53.63 ± 3.46	*p* < 0.05
PW (mm)	11.50 ± 0.5	12.00 ± 0.27	N.S.
cLVM (g)	189.96 ± 17.34	256.14 ± 14.12	*p* < 0.05
IVS (mm)	11.17 ± 0.4	12.50 ± 0.57	N.S.
RVOT EDA (cm^2^)	27.17 ± 1.74	30.00 ± 1.41	*p* < 0.05
RA (cm^2^)	14.05 ± 0.81	14.90 ± 0.93	N.S.

Data are expressed as means ± SEM. Data were compared using independent 2-tailed *t*-test. *p* < 0.05 was considered statistically significant. CABG—coronary artery bypass graft surgery; LVED—left-ventricular end-diastolic diameter; LVES—left-ventricular end-systolic diameter; LA—left-atrial area; LVEF—left-ventricular ejection fraction; PW—thickness of the posterior wall; cLVM—calculated left-ventricular mass; IVS—thickness of interventricular septum; RVOT EDA—right-ventricular outflow tract end-diastolic area; RA—area of the right ventricle; LVH—left-ventricular hypertrophy.

## Data Availability

Data are available from the first author (cziraki.attila@pte.hu).

## References

[1] Nowbar A.N., Gitto M., Howard J.P., Francis D.P., Al-Lamee R. (2019). Mortality from Ischemic Heart Disease. Circ. Cardiovasc. Qual Outcomes.

[2] Sayols-Baixeras S., Lluís-Ganella C., Lucas G., Elosua R. (2014). Pathogenesis of coronary artery disease: Focus on genetic risk factors and identification of genetic variants. Appl. Clin. Genet..

[3] Bhatt A.S., Ambrosy A.P., Velazquez E.J. (2017). Adverse Remodeling and Reverse Remodeling After Myocardial Infarction. Curr. Cardiol. Rep..

[4] Schirone L., Forte M., Palmerio S., Yee D., Nocella C., Angelini F., Pagano F., Schiavon S., Bordin A., Carrizzo A. (2017). A Review of the Molecular Mechanisms Underlying the Development and Progres-sion of Cardiac Remodeling. Oxid. Med. Cell. Longev..

[5] Na H.-M., Cho G.-Y., Lee J.M., Cha M.-J., Yoon Y.E., Lee S.-P., Kim H.-K., Kim Y.-J., Sohn D.-W. (2016). Echocardiographic Predictors for Left Ventricular Remodeling after Acute ST Elevation Myocardial Infarction with Low Risk Group: Speckle Tracking Analysis. J. Cardiovasc. Ultrasound.

[6] Nemeth Z., Cziraki A., Szabados S., Biri B., Keki S., Koller A. (2015). Elevated Levels of Asymmetric Dimethylarginine (ADMA) in the Pericardial Fluid of Cardiac Patients Correlate with Cardiac Hypertrophy. PLoS ONE.

[7] Liu C.-F., Tang W.H.W. (2019). Epigenetics in Cardiac Hypertrophy and Heart Failure. Jacc. Basic Transl. Sci..

[8] Archer C.R., Robinson E., Drawnel F.M., Roderick H.L. (2017). Endothelin-1 promotes hypertrophic remodelling of cardiac myocytes by activating sustained signalling and transcription downstream of endothelin type A receptors. Cell. Signal..

[9] Wang Y., Guo Z., Gao Y., Liang P., Shan Y., He J. (2019). Angiotensin II receptor blocker LCZ696 attenuates cardiac remodeling through the inhibition of the ERK signaling pathway in mice with pregnancy-associated cardiomyopathy. Cell Biosci..

[10] Mandaviya P.R., Stolk L., Heil S.G. (2014). Homocysteine and DNA methylation: A review of animal and human literature. Mol. Genet. Metab..

[11] Kang S.S., Wong P.W.K., Malinow M.R. (1992). Hyperhomocyst(e)inemia as a Risk Factor for Occlusive Vascular Disease. Annu. Rev. Nutr..

[12] Julius U., Pietzsch J., Gromeier S., Schorr H., Herrmann W. (2001). Homocysteine levels in patients treated with lipid apheresis: Effect of a vitamin therapy. Eur. J. Clin. Investig..

[13] Kayadibi H., Sertoglu E., Uyanik M. (2014). Plasma Total Homocysteine Levels in Diabetic Retinopathy. BioMed Res. Int..

[14] Ueland P.M., Refsum H., Stabler S.P., Malinow M.R., Andersson A., Allen R.H. (1993). Total homocysteine in plasma or serum: Methods and clinical applications. Clin. Chem..

[15] Selhub J., Jacques P.F., Bostom A.G., D’Agostino R.B., Wilson P.W.F., Belanger A.J., O’Leary D.H., Wolf P.A., Schaefer E.J., Rosenberg I.H. (1995). Association between Plasma Homocysteine Concentrations and Extracranial Carot-id-Artery Stenosis. N. Engl. J. Med..

[16] Humphrey L.L., Fu R., Rogers K., Freeman M., Helfand M. (2008). Homocysteine Level and Coronary Heart Disease Incidence: A Systematic Review and Me-ta-analysis. Mayo Clin. Proc..

[17] Milani R.V., Lavie C.J. (2008). Homocysteine: The Rubik’s Cube of Cardiovascular Risk Factors. Mayo Clin. Proc..

[18] Ganguly P., Alam S.F. (2015). Role of homocysteine in the development of cardiovascular disease. Nutr. J..

[19] Koller A., Szenasi A., Dornyei G., Kovacs N., Lelbach A., Kovacs I. (2018). Coronary Microvascular and Cardiac Dysfunction Due to Homocysteine Pathometabolism; A Complex Therapeutic Design. Curr. Pharm. Des..

[20] Joseph J., Joseph L., Shekhawat N.S., Devi S., Wang J., Melchert R.B., Hauer-Jensen M., Kennedy R.H. (2003). Hyperhomocysteinemia leads to pathological ventricular hypertrophy in normotensive rats. Am. J. Physiol. Circ. Physiol..

[21] Velazquez E.J., Lee K.L., Jones R.H., Al-Khalidi H.R., Hill J.A., Panza J.A., Michler R.E., Bonow R.O., Doenst T., Petrie M.C. (2016). Coronary-Artery Bypass Surgery in Patients with Ischemic Cardiomyopathy. New Engl. J. Med..

[22] Ottani F., Galvani M., Nicolini F.A., Ferrini D., Pozzati A., Di Pasquale G., Jaffe A.S. (2000). Elevated cardiac troponin levels predict the risk of adverse outcome in patients with acute coronary syndromes. Am. Hear. J..

[23] Shah A.S.V., Anand A., Sandoval Y., Lee K.K., Smith S.W., Adamson P.D., Chapman A.R., Langdon T., Sandeman D., Vaswani A. (2015). High-sensitivity cardiac troponin I at presentation in patients with suspected acute coronary syndrome: A cohort study. Lancet.

[24] Hessel M.H.M., Atsma D.E., van der Valk E.J.M., Bax W.H., Schalij M.J., van der Laarse A. (2008). Release of cardiac troponin I from viable cardiomyocytes is mediated by in-tegrin stimulation. Pflugers Arch..

[25] Storti S., Prontera C., Parri M.S., Iervasi A., Vittorini S., Emdin M., Zucchelli G.C., Longombardo G., Migliorini P., Clerico A. (2006). Evaluation of the analytical performance of the advanced method for cardiac troponin I for the AxSYM platform: Comparison with the old method and the Access system. Clin. Chem. Lab. Med..

[26] Lonati S., Novembrino C., Ippolito S., Accinni R., Galli C., Troonen H., Campolo J., Della Noce C., Lunghi G., Bamonti Catena F. (2004). Analytical performance and method comparison study of the total homocysteine fluorescence polarization immunoassay (FPIA) on the AxSYM analyzer. Clin. Chem Lab. Med..

[27] Otterstad J.E. (2002). Measuring left ventricular volume and ejection fraction with the biplane Simpson’s method. Heart.

[28] Devereux R.B., Alonso D.R., Lutas E.M., Gottlieb G.J., Campo E., Sachs I., Reichek N. (1986). Echocardiographic assessment of left ventricular hypertrophy: Comparison to necropsy findings. Am. J. Cardiol..

[29] Yeh J.-K., Chen C.-C., Hsieh M.-J., Tsai M.-L., Yang C.-H., Chen D.-Y., Chang S.-H., Wang C.-Y., Lee C.-H., Hsieh I.-C. (2017). Impact of Homocysteine Level on Long-term Cardiovascular Outcomes in Patients after Coronary Artery Stenting. J. Atheroscler. Thromb..

[30] Bagi Z., Csekö C., Tóth E., Koller A. (2003). Oxidative stress-induced dysregulation of arteriolar wall shear stress and blood pressure in hyperhomocysteinemia is prevented by chronic vitamin C treatment. Am. J. Physiol. Circ. Physiol..

[31] Sundström J., Sullivan L., Selhub J., Benjamin E.J., D’Agostino R.B., Jacques P.F., Rosenberg I.H., Levy D., Wilson P.W., Vasan R.S. (2004). Relations of plasma homocysteine to left ventricular structure and function: The Framingham Heart Study. Eur. Hear. J..

[32] Chen F., Guo Y.-H., Gao W., Feng X.-H., Chen L., Tang C.-S. (2006). [Effect of hyperhomocysteinemia on cardiac remodeling in rats]. Beijing Da Xue Xue Bao Yi Xue Ban = J. Peking Univ. Heal. Sci..

[33] Ungvari Z., Csiszar A., Edwards J.G., Kaminski P.M., Wolin M.S., Kaley G., Koller A. (2003). Increased superoxide production in coronary arteries in hyperhomocysteinemia: Role of tumor necrosis factor-alpha, NAD(P)H oxidase, and inducible nitric oxide synthase. Arterioscler. Thromb. Vasc. Biol..

[34] Nemeth Z., Cziraki A., Szabados S., Horvath I., Koller A. (2015). Pericardial fluid of cardiac patients elicits arterial constriction: Role of endothelin-1. Can. J. Physiol. Pharmacol..

[35] Veresh Z., Debreczeni B., Hamar J., Kaminski P.M., Wolin M.S., Koller A. (2012). Asymmetric Dimethylarginine Reduces Nitric Oxide Donor-Mediated Dilation of Arterioles by Activating the Vascular Renin-Angiotensin System and Reactive Oxygen Species. J. Vasc. Res..

[36] Cziraki A., Lenkey Z., Sulyok E., Szokodi I., Koller A. (2020). L-Arginine-Nitric Oxide-Asymmetric Dimethylarginine Path-way and the Coronary Circulation: Translation of Basic Science Results to Clinical Practice. Front. Pharmacol..

[37] Cohn J.N., Ferrari R., Sharpe N., on Behalf of an International Forum on Cardiac Remodeling (2000). Cardiac remodeling—concepts and clinical implications: A consensus paper from an inter-national forum on cardiac remodeling. J. Am. Coll. Cardiol..

[38] Czubryt M.P. (2019). Cardiac Fibroblast to Myofibroblast Phenotype Conversion—An Unexploited Therapeutic Target. J. Cardiovasc. Dev. Dis..

[39] Bagchi R.A., Roche P., Aroutiounova N., Espira L., Abrenica B., Schweitzer R., Czubryt M.P. (2016). The transcription factor scleraxis is a critical regulator of cardiac fibroblast phenotype. BMC Biol..

[40] Kanisicak O., Khalil H., Ivey M.J., Karch J., Maliken B.D., Correll R.N., Brody M.J., Lin S.-C.J., Aronow B.J., Tallquist M.D. (2016). Genetic lineage tracing defines myofibroblast origin and function in the injured heart. Nat. Commun..

[41] Vallée A., Lecarpentier Y. (2019). TGF-β in fibrosis by acting as a conductor for contractile properties of myofibroblasts. Cell. Biosci..

[42] Duong T.E., Hagood J.S. (2018). Epigenetic Regulation of Myofibroblast Phenotypes in Fibrosis. Curr. Pathobiol. Rep..

[43] Bode-Böger S.M., Scalera F., Ignarro L.J. (2007). The l-arginine paradox: Importance of the l-arginine/asymmetrical dimethylarginine ra-tio. Pharmacol. Ther..

[44] Suematsu N., Ojaimi C., Kinugawa S., Wang Z., Xu X., Koller A., Recchia F.A., Hintze T.H. (2007). Hyperhomocysteinemia Alters Cardiac Substrate Metabolism by Impairing Nitric Oxide Bioavailability Through Oxidative Stress. Circulation.

[45] Okuyan E., Uslu A., Çakar M.A., Sahin I., Önür I., Enhos A., Biter H.A., Çetin Ş., Dinçkal M.H. (2010). Homocysteine Levels in Patients with Heart Failure with Preserved Ejection Fraction. Cardiology.

[46] Gupta A., Moustapha A., Jacobsen D.W., Goormastic M., Tuzcu E.M., Hobbs R., Young J., James K., McCarthy P., van Lente F. (1998). High Homocysteine, Low Folate, and Low Vitamin B6 Concentrations: Prevalent Risk Factors for Vascular Disease in Heart Transplant Recipients. Transplantation.

[47] Williams B., Mancia G., Spiering W., Agabiti Rosei E., Azizi M., Burnier M., Clement D.L., Coca A., de Simone G., Dominiczak A. (2018). 2018 ESC/ESH Guidelines for the management of arterial hypertension. Eur. Heart J..

[48] Fu Y.N., Xiao H., Ma X.W., Jiang S.Y., Xu M., Zhang Y.Y. (2011). Metformin attenuates pressure overload-induced cardiac hy-pertrophy via AMPK activation. Acta Pharmacol. Sin..

[49] Li J., Minćzuk K., Massey J.C., Howell N.L., Roy R.J., Paul S., Patrie J.T., Kramer C.M., Epstein F.H., Carey R.M. (2020). Metformin Improves Cardiac Metabolism and Function, and Prevents Left Ventricular Hypertrophy in Spontaneously Hypertensive Rats. J. Am. Hear. Assoc..

[50] Mohan M., Al-Talabany S., McKinnie A., Mordi I.R., Singh J.S.S., Gandy S.J., Baig F., Hussain M.S., Bhalraam U., Khan F. (2019). A randomized controlled trial of met-formin on left ventricular hypertrophy in patients with coronary artery disease without diabetes: The MET-REMODEL trial. Eur. Heart J..

[51] Zhang Q., Li S., Li Q., Ren K., Sun X., Li J. (2016). Metformin Treatment and Homocysteine: A Systematic Review and Meta-Analysis of Randomized Controlled Trials. Nutrients.

[52] Aung N., Sanghvi M.M., Piechnik S.K., Neubauer S., Munroe P.B., Petersen S.E. (2020). The Effect of Blood Lipids on the Left Ventricle: A Mendelian Randomization Study. J. Am. Coll. Cardiol..

[53] Liao J.K. (2004). Statin therapy for cardiac hypertrophy and heart failure. J. Investig. Med..

[54] Wile D.J., Toth C. (2010). Association of Metformin, Elevated Homocysteine, and Methylmalonic Acid Levels and Clinically Wors-ened Diabetic Peripheral Neuropathy. Diabetes Care.

